# Effects of Buprenorphine Dose and Therapeutic Engagement on Illicit Opiate Use in Opioid Use Disorder Treatment Trials

**DOI:** 10.3390/ijerph19074106

**Published:** 2022-03-30

**Authors:** Andrew W. Bergen, James W. Baurley, Carolyn M. Ervin, Christopher S. McMahan, Joe Bible, Randall S. Stafford, Seshadri C. Mudumbai, Andrew J. Saxon

**Affiliations:** 1 Oregon Research Institute, Eugene, OR 97403, USA; 2 BioRealm, LLC, Walnut, CA 91789, USA; baurley@biorealm.ai (J.W.B.); ervin@biorealm.ai (C.M.E.); 3 School of Mathematical and Statistical Sciences, Clemson University, Clemson, SC 29634, USA; mcmaha2@clemson.edu (C.S.M.); jdbible@clemson.edu (J.B.); 4 Department of Medicine, Stanford University, 300 Pasteur Drive, Stanford, CA 94305, USA; rstafford@stanford.edu; 5 Anesthesia Service, VA Palo Alto Health Care System, 3801 Miranda Avenue, Palo Alto, CA 94304, USA; mudumbai@stanford.edu; 6 Department of Anesthesiology, Perioperative, and Pain Medicine, School of Medicine, Stanford University, Stanford, CA 94305, USA; 7 Center of Excellence in Substance Addiction Treatment and Education, VA Puget Sound Health Care System, Seattle, WA 98108, USA; asaxon@uw.edu; 8 Department of Psychiatry and Behavioral Sciences, University of Washington, Seattle, WA 98195, USA

**Keywords:** opioid-related disorders, buprenorphine, meta-analysis, opiate substitution treatment, urinalysis

## Abstract

The impact of agonist dose and of physician, staff and patient engagement on treatment have not been evaluated together in an analysis of treatment for opioid use disorder. Our hypotheses were that greater agonist dose and therapeutic engagement would be associated with reduced illicit opiate use in a time-dependent manner. Publicly-available treatment data from six buprenorphine efficacy and safety trials from the Federally-supported Clinical Trials Network were used to derive treatment variables. Three novel predictors were constructed to capture the time weighted effects of buprenorphine dosage (mg buprenorphine per day), dosing protocol (whether physician could adjust dose), and clinic visits (whether patient attended clinic). We used time-in-trial as a predictor to account for the therapeutic benefits of treatment persistence. The outcome was illicit opiate use defined by self-report or urinalysis. Trial participants (N = 3022 patients with opioid dependence, mean age 36 years, 33% female, 14% Black, 16% Hispanic) were analyzed using a generalized linear mixed model. Treatment variables dose, Odds Ratio (OR) = 0.63 (95% Confidence Interval (95%CI) 0.59–0.67), dosing protocol, OR = 0.70 (95%CI 0.65–0.76), time-in-trial, OR = 0.75 (95%CI 0.71–0.80) and clinic visits, OR = 0.81 (95%CI 0.76–0.87) were significant (*p*-values < 0.001) protective factors. Treatment implications support higher doses of buprenorphine and greater engagement of patients with providers and clinic staff.

## 1. Introduction

Over 10 million adolescents and adults in the United States were estimated to have misused prescription analgesics and/or used heroin in 2019, and 1.6 million were estimated to have a past year opioid use disorder (OUD) [1]. Medication for OUD treatment (MOUD) in the United States includes three Food and Drug Administration-approved medications combined with psychosocial and supportive therapies [2]. The medications bind to the mu opioid receptor (MOR), and include methadone (approved 1972), naltrexone (approved 1984), and buprenorphine (alone or in combination with naloxone, approved 2002). The approval of buprenorphine and buprenorphine–naloxone in the United States followed decades of translational research involving clinicians, scientists, and policy-makers in public agencies, medical institutes, a pharmaceutical company, and treatment clinics [3].

Researchers in the Addiction Research Center of the National Institute of Drug Abuse (NIDA) and the Behavioral Pharmacology Research Unit of Johns Hopkins University performed early clinical studies. Buprenorphine and morphine were compared in patients with heroin dependence, with buprenorphine demonstrating lower intrinsic activity than morphine, and a blockade of morphine effects [4]. Buprenorphine and methadone were compared in a randomized clinical trial (RCT) detoxification protocol in patients with heroin dependence, with no significant differences in retention, illicit drug use or reported symptoms observed, though methadone exerted a greater blockade of hydromorphone effects [5]. Buprenorphine and two doses of methadone were evaluated in an RCT, with buprenorphine and the higher methadone dose exhibiting greater retention, lower urinalysis and lower relapse rates than the lower methadone dose [6].

Federally-supported research addressed effectiveness in diverse settings and populations, medication formulation and delivery, tapering, safety and effectiveness in patients with heroin and with prescription opioid dependence [7]. These RCTs took place in community-based clinics, coordinated by the Veterans Affairs Cooperative Studies Program and the National Drug Abuse Treatment Clinical Trials Network. Results from these RCTs were critical to clinically validate buprenorphine MOUD as an MOR partial agonist with lower risk for dependence, effective in preventing opioid abstinence syndrome, and able to be initiated in an office setting and self-administered at home [7]. Legislative, policy and regulatory innovations were necessary to enable physicians to treat patients with buprenorphine in the office-based setting [8]. Additional legislation enabled physician assistants and nurse practitioners to be waivered to prescribe buprenorpine and increased patient limits, but multiple payor, practice and provider challenges have limited the number of patients receiving buprenorphine MOUD [9].

In a multiple healthcare system administrative dataset analysis of over 1 M patients (2013–2016), OUD prevalence was 1%, and 21% of patients with OUD were treated with buprenorphine [10]. Slightly higher rates of MOUD (28%) were observed across Veterans Health Administration (VHA) residential treatment clinics (2012), with provider and staff education and engagement associated with higher rates [11]. A provider MOUD education initiative [12] in VHA non-addiction speciality clinics showed significant increases in proportions of OUD patients prescribed MOUD, and of clinicians prescribing MOUD (mostly buprenorphine) post-initiative [13]. However, MOUD retention rate was lower in education initiative clinic patients than in comparison clinic patients.

Clinical practice guidelines on buprenorphine MOUD provide guidance on clinical assessment, the establishment of a treatment contract, stages of treatment (induction, stabilization and maintenance), psychosocial treatment, adherence, and comorbidities and concomitant treatments [14]. The earliest practice guidelines emphasized the importance of clinical judgement with respect to induction, dosing, and of necessary concomitant psychosocial treatment [8,15,16]. Dosing, monitoring and duration of buprenorphine MOUD remains clinically complex due to individual patient factors [17,18,19].

As part of a research program to understand predictors of MOUD success, we harmonized and analyzed publicly available individual participant data from efficacy and safety trials of buprenorphine treatment. We developed an initial model to account for potential time dependent effects of treatment using the clinical practice of daily opioid substitution dosing as an exemplar. We used a generalized linear mixed model approach to estimate the time dependent therapeutic effects of buprenorphine dose [18,20], clinician judgement with respect to buprenorphine dose [21], the clinic environment [22], and persistence in treatment [23] for illicit opiate use. We identified and ranked multiple significant treatment effects which emphasize the importance of dose and the therapeutic engagement of providers and patients. Our results provide a novel perspective on the complexity of factors influencing buprenorphine treatment success.

## 2. Materials and Methods

### 2.1. Trial Data, Informed Consent and Ethical Approval

We searched NIDA Data Share using the keyword “opiate”; the keyword “buprenorphine” identifies the same RCTs. We selected buprenorphine efficacy and safety treatment RCTs for opioid use disorder for secondary data analysis from the NIDA’s Data Share resource (datashare.nida.nih.gov, accessed on 5 September 2017). These RCTs (NIDA Data Share and clinicaltrials.gov, accessed on 5 September 2017, NCT identification codes) were: NIDA-CSP-999 and NCT00000207 [24]; NIDA-CSP-1008A and NCT00015028 [25]; NIDA-CSP-1008B and NCT00015171 [25]; NIDA-CSP-1018 and NCT00007527 [26]; NIDA-CTN-0027 and NCT00315341 [27]; and, NIDA-CTN-0030 and NCT00316277 [28]. We excluded RCTs that were primarily designed to assess buprenorphine effectiveness in opioid detoxification, short-term tapering, treating adolescent patients, treating patients with comorbid cocaine and opioid dependence primarily for cocaine use outcomes, were long-term follow-up studies without treatment, or were released after the project proposal was submitted (September 2017). Informed consent was obtained by the study coordinators and clinical investigators of the treatment trials from all trial participants; however, informed consent forms are not available on datashare.nida.nih.gov, accessed on 5 September 2017. Trial data were systematically deidentified before being made available (see deidentification notes on datashare.nida.nih.gov, accessed on 5 September 2017). An exemption from Institutional Review Board (IRB) review was obtained from the IRB of BioRealm LLC (IRB ID: 5610).

#### Trial Design and Ascertainment Criteria

Safety, efficacy or both were the design objectives of five trials; counseling efficacy was the design objective of one trial (Table S1). There were nine treatment phases in the six trials, seven of nine phases included flexible (per clinician judgement) dosing. The range of behavioral counseling was 0.25–1.25 h per protocol in eight phases, with one phase relying upon usual clinic practice. Urinalysis was the primary or secondary outcome in five trials, while self-reported lapse was the outcome in one trial.

Inclusion criteria (Table S2) included current opioid dependence by DSM-IV criteria, adult (one trial evaluated a small number of 15–18 year old individuals for detoxification with continued maintenance treatment determined by clinical judgement) male and non-pregnant/non-breastfeeding female participants. Participants were required to be in good physical health or in the care of a physician, to be agreeable to study conditions and able to give informed consent. Exclusion criteria (Tables S3 and S4) were more numerous, but consistently excluded individuals with any acute medical condition that would make participation medically hazardous in the clinical judgement of the study investigator/physician, any current substance use dependence diagnosis excluding opiate, caffeine and nicotine dependence, expected inability to complete the study and refusal to use birth control if of childbearing potential. Three trials excluded potential participants with: current severe psychiatric conditions; current alcohol, sedative or stimulant dependence requiring immediate medical attention; recent investigational drug study participation; or recent or discontinued treatment with opioid agonists or naltrexone for opioid dependence. Five trials excluded participants with elevated liver enzymes, or recent participation in a methadone maintenance treatment program.

### 2.2. Statistical Analyses

#### 2.2.1. Predictors and Outcome

We estimated the effects of buprenorphine MOUD treatment variables on short term lapse, the outcome—defined as a positive participant urinalysis (≥300 nanogram/milliliter, “Opiate 300”), and in two trials with more extensive testing, any positive opiate or opioid test) or self-reported illicit opiate use at the clinic visit in question without reference to prior negative or positive tests or self-reports. Our analytic strategy enabled estimation of the effects of multiple treatment variables including adjustment for numerous participant covariates.

#### 2.2.2. Trial Variable Harmonization

We performed variable harmonization using publicly available trial protocols, data dictionaries, and patient-level data, in a systematic step-wise fashion. We grouped variables by domain and cataloged the information available from each trial. Domains included sociodemographics, methadone treatment history, drug use history, route of administration, substance use disorder and psychiatric diagnoses, buprenorphine dosage, clinical visits, and illicit opiate use (self-reported and urinalysis). We matched the variables across the trials and for select variables, inspected the coding, labels, and distributions in each trial. We created new harmonized variables across trials. Clinical expert consultation occurred twice during harmonization.

#### 2.2.3. Treatment Variables

Treatment variables were derived from trial protocols and from patient data on daily dose and clinic visits. We harmonized four treatment related variables across trials: dose (mg buprenorphine/day); adaptive dose (whether dose was fixed by protocol or could be modified by clinical judgement); clinic visit (whether the patient self-administered the daily dose at home or in clinic under observation); and time-in-trial (trial day urinalysis or self-report occurred).

We hypothesized that treatment effects on illicit opiate use depend on treatments received prior to the day of the urinalysis or self-report. The extended plasma half-life and duration of action of sublingually-administered buprenorphine [20,29] supports a time dependent approach to analysis of dose. The clinical hypotheses that provider and patient engagement have positive effects on treatment outcomes [30], that clinically supervised administration may promote therapeutic engagement [22], and that patient distress tolerance may influence abstinence [23] or improve with abstinence [31], support analyses of the effects of therapeutic engagement and retention in treatment on MOUD treatment outcomes, here, short-term lapse.

We implemented these treatment hypotheses using three derived variables (Time Weighted Dose, Time Weighted Adaptive Dose, and Time Weighted Clinic Visit) and one assigned variable (Time-in-Trial). For the time-weighted variables, at the *j*th clinic visit occurring at time $t_{j}$, the value was calculated using all previous (daily) values with weights of ${\left(1/2\right)}^{t}$, for $t=0,\cdots ,t_{j}-2$, with the more recent values of the variables being given higher weights (lower exponents). That is, the time-weighted variables take into account past treatment, discounted by 50% for each dose period. Thus, let $D_{it}$ denote the dose level taken by the *i*th subject at time *t*, the time-weighted dose at $t_{j}$ is equal to:

(1) $$\begin{array}{c} \sum_{t=1}^{t_{j}-1}{\left(1/2\right)}^{t_{j}-t-1}D_{it}/\sum_{t=1}^{t_{j}-1}{\left(1/2\right)}^{t_{j}-t-1}. \end{array}$$

In practice, the Time Weighted Dose for a participant who is completely adherent to a protocol or clinician assigned dose will be the same as the assigned dose. Thus, a participant assigned a daily dose of 16 milligram (mg) of buprenorphine will have a Time Weighted Dose of 16 mg if the patient self-administers 16 mg every day. The time dependent coding of Time Weighted Dose directly accounts for adherence or non-adherence up to the present day. For example, consider two participants assigned to a 16 mg dose arm who are adherent the first week of the trial, but each miss one dose in the second week of the trial. One participant misses their dose on the first day of the second week and their Time Weighted Dose on the 14th day of the trial would be 15.75 mg. The other participant misses their dose on the sixth day of the second week and their Time Weighted Dose on the 14th day of the trial would be 7.99 mg.

For the Time Weighted Adaptive Dose and Time Weighted Clinic Visit variables, the underlying treatment data are binary (0 or 1). For these variables, non-adherence occurs when the patient does not attend a scheduled clinic visit. On such a day, there is no opportunity for patient–clinician engagement and a potential dose adjustment (Adaptive Dose on the day = 0), or for patient–clinical staff engagement and the therapeutic benefits associated with self-administration under observation (Clinic Visit on the day = 0).

Time-in-trial was the fourth time-dependent treatment variable; this variable was assigned using the number of days that have occurred in the trial on the day of each urinalysis or self-report.

#### 2.2.4. Patient Covariates

After filtering candidate covariates with missingness >25%, we imputed missing data using the regularized iterative factorial analysis for a mixed data algorithm [32].

#### 2.2.5. Generalized Linear Mixed Model

Define $Y_{ij}$, for $j=1,\cdots ,n_{i}$ and i=1,⋯,m, to be a binary indicator, such that $Y_{ij}=1$ denotes the event that the *i*th participant tests positively for opiates during the *j*th visit and $Y_{ij}=0$ otherwise. To relate predictors and covariates to the binary response, we posit the following generalized linear mixed model:

(2) $$\begin{array}{c} g\left\{P\left(Y_{ij}=1|X_{ij}\right)\right\}=X_{ij}^{\prime }\beta +\gamma_{0i}+\gamma_{1k(i)}, \end{array}$$

where g(·) is the logistic link, $X_{ij}$ is a vector of covariate information, β is a vector of predictor coefficients, $\gamma_{0i}$ is a participant specific random effect specified to account for heterogeneity across subjects, $\gamma_{1k(i)}=\gamma_{1k}$ if the *i*th participant is a part of the *k*th trial, for k=1,⋯,K, and $\gamma_{1k}$ is a random effect specified to account for the heterogeneity across trials. The random effects distributions were taken to be:

(3) $$\begin{array}{cc} \gamma_{0i} & \overset{iid}{∼}N\left(0,\sigma_{0}^{2}\right) \end{array}$$

(4) $$\begin{array}{cc} \gamma_{1k} & \overset{iid}{∼}N\left(0,\sigma_{1}^{2}\right). \end{array}$$

Note that the random effects are specified to be independent given their nesting. For computational stability, all quantitative variables were standardized to have a mean of zero and a standard deviation of one. Model fitting was completed via PROC MIXED in SAS version 9.4 (Cary, NC, USA).

#### 2.2.6. Assessment of Results

A *p*-value < 0.001 was considered statistically significant based on the following logic: 58 variable effect estimates were estimated in 22 treatment, sociodemographic and drug use domains; a Bonferroni correction (0.05/58=0.00086) is conservative, thus, we chose α<0.001, which is slightly less conservative.

## 3. Results

### 3.1. Participant Characteristics

There were 3022 participants after selection of those randomized and enrolled into buprenorphine or placebo arms from the 4853 screened individuals (Table S5). After filtering for missingness, there were 22 candidate risk factors (four treatment, nine sociodemographic and nine drug use history), with 58 variable effects to be estimated.

#### 3.1.1. Sociodemographics

The mean (Standard Deviation, SD) age of participants was 36.1 (9.8) years, one-third (33%) self-identified as female and over one-third (35%) self-identified as one of five minority Office of Management and Budget race or ethnicity categories. Nearly half (48%) of the participants had a high school education, while over one-third (37%) had completed higher levels of education. Two-thirds (66%) of participants had full time or part time employment, while half (52%) and one quarter (26%) had blue and white collar employment histories, respectively. Approximately one-third of participants were either married (35%), never-married (34%), or widowed, separated or divorced (30%). Most participants (92%) lived with a child, friends, parents, partner or partner and child. See Table S7.

#### 3.1.2. Drug Use History

All trial participants fulfilled opiate or prescription opioid dependence criteria. Most (78%) participants reported a heroin use history. Participants reported using illicit opiates or misusing prescription analgesic opioids for a mean (SD) of 8.2 (8.4) years of use. The common modes of opiate or opioid use were intravenous (56%) and nasal (36%). Non-opiate/opioid drug use history was common. Nearly two-thirds of participants reported the use of marijuana, alcohol or cocaine, one-third the use of tranquilizers, one-quarter the use of methamphetamines, and one-sixth the use of phencyclidine. See Table S7.

### 3.2. Treatment Variables and Short-Term Lapse

#### 3.2.1. Dose Records and Urinalysis or Self-Report Records

There were N = 479,141 dose records (mg buprenorphine) across the 3022 participants used to code time weighted values for the two dose related variables and the clinic visit variable. There were 55,739 urinalysis or self-report records across the 3022 participants. The four treatment variables were each assigned 55,739 values across the 3022 participants. These 55,739 values were used in the regression analysis to estimate treatment variable effects.

#### 3.2.2. Treatment and Outcome Characteristics

Descriptive statistics for the four unstandardized treatment variables and the outcome variable are presented in Table 1.

**Figure 1:**
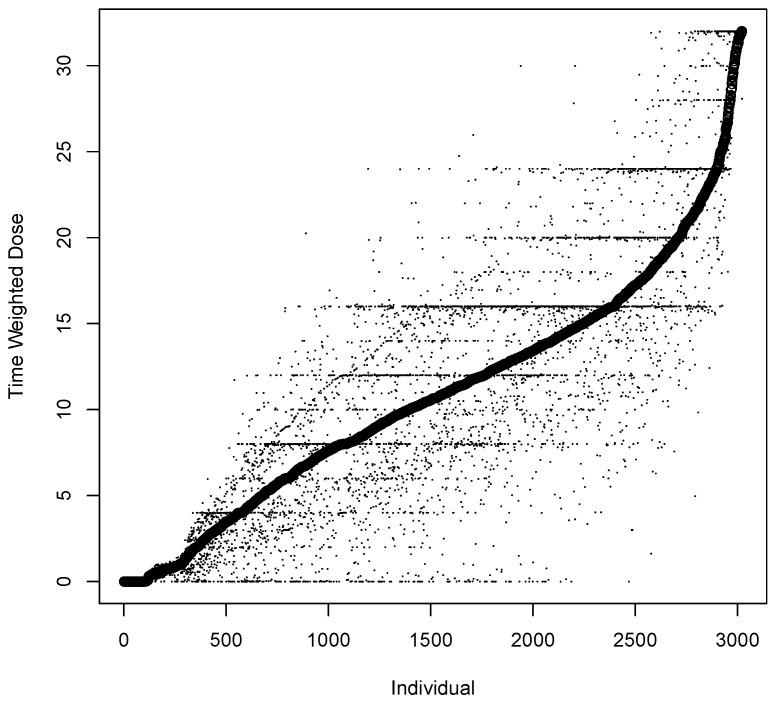
Mean Time Weighted Dose from 0 to 32 mg. Dots are participant 25th and 75th quantiles.

The descriptive statistics in Table 1 provide immediate insight into the characteristics of time dependent treatment variables and of short-term lapse in the analytic sample. The distribution of the unstandardized mean Time Weighted Dose is plotted in Figure 1; each set of points on the x-axis represents a participant while the y-axis presents the 25%, the mean and the 75% of each participant. Figure 1 demonstrates the variability of Time Weighted Dose across and within participants. While mean Time Weighted Dose extended from zero (fixed dose placebo arms) to the maximal recommended dose of 32 mg, the overall mean and median are at 11.9 mg and 12 mg, and the number of participants with mean Time Weighted Dose values from 24 to 32 mg is less than the number of participants in 8 mg dose ranges below 24 mg.

The mean and median values of unstandardized Time Weighted Adaptive Dose (0.76 and 1.00) reflect seven trial phases with flexible dosing and two trial phases with fixed doses, that is, most doses were assigned under a flexible dosing protocol. The mean and median of unstandardized Time Weighted Clinic Visit indicate that most doses were self-administered at home. The distribution of unstandardized Time-in-Trial reflects the wide range of participant treatment engagement with most individuals leaving treatment three months after treatment initiation, even though five trials provided treatment for eight or 12 months and three provided extended treatment protocols. The mean and median of Short-term Lapse indicates that a minority of urinalysis assays or self-reports indicated use or misuse of illicit or prescription opioids.

**Figure 2:**
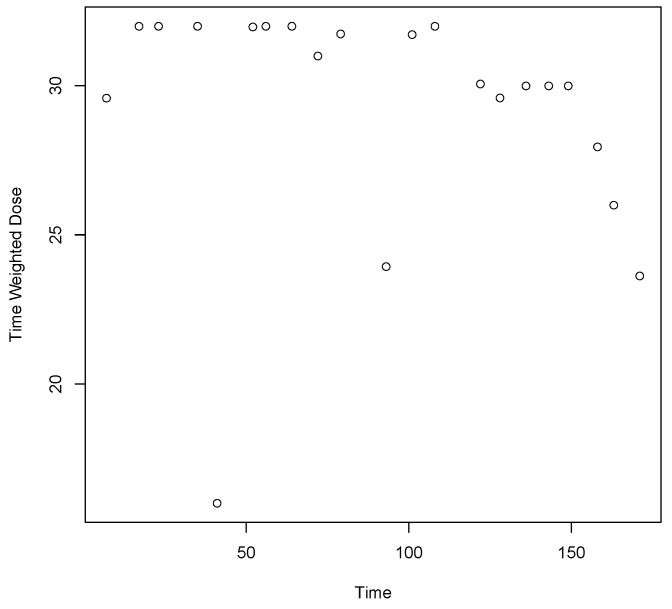
Time Weighted Dose of a randomly selected trial participant by days in trial. See Table S8 for daily mg dose, urinalysis days and Time Weighted Dose. The participant missed doses on days 40, 41, 67, 72, 86 and 93. The minimum Time Weighted Dose is on day 41. The maximum Time Weighted Dose is on day 35.

The Time Weighted Dose of a randomly selected participant is plotted in Figure 2 to illustrate the pattern of variation of this derived time dependent variable. The daily mg buprenorphine dose, urinalysis days and Time Weighted Dose data from this participant are found in Table S8. This participant missed doses on days 40 and 41, days 67 and 72, and days 86 and 93. The missed doses had the effect of reducing Time Weighted Dose on days 41, 72, and 93. The minimum derived value (16.00 mg at day 41) is the result of the missed doses on days 40 and 41, while the next minimum Time Weighted Dose (23.63 at day 171) is due to the tapering doses at the end of the trial. The maximum derived value is 32.00 mg on day 35, that is, it is reached early in the trial before missing doses are recorded; however, there are multiple values later in the trial that approach 32 mg.

### 3.3. Results from the Generalized Linear Mixed Model Analysis

We identified statistically significant (*p* < 0.001) protective effects associated with each of the four time dependent treatment variables on Short-term Lapse (Table 2). The rank order of the treatment variable effect sizes was Time Weighted Dose > Time Weighted Adaptive Dose > Time-in-Trial > Time Weighted Clinic Visit.

#### Participant Factors

We also identified participant covariates significantly (*p* < 0.001) associated with Short-term Lapse. We name these factors with their direction, but do not tabulate details as our focus is on the time-dependent treatment variables. Older age and an executive work role reduced the risk of Short-term Lapse, while a marital status of separated, and living alone or in an unstable arrangement increased risk. No history of heroin use and an oral mode of opioid administration reduced risk of Short-term Lapse, while a tranquilizer use history increased risk.

## 4. Discussion

We investigated the influence of four treatment variables on short-term lapse, using publicly available individual participant data from buprenorphine treatment trials. We discuss findings and limitations, and the clinical and research implications of our findings below.

### 4.1. Treatment Factor Findings

Time-weighted dose of buprenorphine was the most protective treatment factor, reflecting the efficacy of buprenorphine for MOUD [7]. The finding that increased dose was protective in CTN treatment trials is supported by laboratory studies that show increased buprenorphine doses increase plasma levels, reduce MOR availability and decrease withdrawal symptoms [33], and by prolonged adherence to greater doses in a prescription drug monitoring program analysis [34]. Our conceptualization of time-weighted dose is an initial model for recent effects of buprenorphine dose and treatment adherence. However, the formal time-weighting model is a hypothesis and requires refinement, for example, using more sophisticated approaches to estimate dose effects over time.

The protective effect of time-weighted adaptive dose, the second ranked treatment factor, reflects the therapeutic benefits of patient and clinician interactions in their assessment of treatment progress and clinical judgement, an essential part of buprenorphine treatment. We recognize that the trials with fixed dose protocols had some fixed doses in the lower half of the recommended dose range before permitting adaptive dosing [24,25], which may have influenced effect estimation. Interaction analysis of time weighted dose and adaptive dose treatment variables might better characterize the influence of each factor.

The third ranked treatment variable, time-in-trial, is usually analyzed as an outcome variable, that is, retention is usually conceptualized as an effect of treatment, for example, in one of the randomized trials in this analysis, participants randomized to methadone exhibited better retention than participants randomized to buprenorphine [35]. In our analysis, we used time-in-trial as a predictor of short-term lapse, a novel application. Analyses of Massachusetts Medicaid administrative data identified MOUD episode length as significantly protective against proxies for relapse [36]. The modeling of participant behavioral measures assessed through treatment may help identify moderators of time-in-trial analyzed as a predictor [23,31].

Effects of the fourth ranked time-weighted clinic visit variable may reflect patient interactions with clinic staff and environment while being observed self-administering a dose. Future analyses of the influence of clinical environments and interactions with staff including tele-health will require new measures and models [37,38].

### 4.2. Limitations

Harmonization was limited by different eligibility criteria and the differences in collection or coding of some variables, across trials. Therefore, we focused harmonization and analysis efforts on variable domains with data available across all trials. Thus, we did not perform subset analyses to estimate the effects of important covariates such as psychiatric comorbidity or chronic pain, common among patients with OUDs [39,40]. We did include a participant specific random effect for all participants which would account for the effects of such comorbidities. Subset analyses of these trials would provide an estimate of the effect of these important factors. In our analyses, we specified the random effects to be independent. It is possible to estimate models under more general correlation structures. We do not expect that primary conclusions would be altered under different random effect specifications.

A general limitation of our analyses is that we did not model the potential influence of per protocol or as delivered behavioral therapy. However, as clinic visits were at the heart of our derived variables, and because behavioral therapy was delivered during clinic visits, the Time Weighted Clinic Visit variable may have incorporated some behavioral therapy effects, and the random effect variable for trial may have accounted for some behavioral therapy effect differences as behavioral therapy protocols differed modestly between trials. Another general limitation is that current drug dependence diagnoses (excluding caffeine, opioid and tobacco dependence) were exclusion criteria.

### 4.3. Treatment Implications

The four novel time dependent treatment variables assessed in this analysis illustrate the complexity of pharmacologic and clinical factors involved in response to MOUD. In this sample, overall positive urinalysis was common at 41%, and half of the participants had left treatment by three months, representing room to improve MOU outcomes. The implications of treatment variable effect estimates are quantitative and qualitative.

- The range of mean time weighted dose in clinical trial participants analyzed covers the range of recommended daily doses [14] and the overall sample mean time weighted dose of 11.94 mg (that incorporates non-adherence) is just under the mean daily buprenorphine dose of 13.4 mg reported in 2015–2018 by N = 1105 physicians treating OUD patients characterized as stable patients [41]. This suggests that, despite some differences in patient characteristics, the clinical trial participant finding that increased buprenorphine dose decreased short-term lapse may apply to current office-based patient practice.
- The protective effects of adaptive dosing and the range of time weighted dose support the understanding that buprenorphine MOUD is a highly personalized therapy [18,19].
- Time-in-trial may represent effects of both patient characteristics and the influence of successful treatment over time [42]. Future analyses to understand time-in-trial associations with short term lapse at different times during treatment may help distinguish patient and treatment related factors influencing time-in-trial association with response to MOUD. Until then, efforts to support retention should result in better response to MOUD.
- In the same study reporting on office-based treatment practice [41], physicians reported they were likely to increase the frequency of office visits in response to all (N = 16) patient vignettes presented. In the same study, most (12/16) patient vignettes elicited scores suggesting no change of dose and only one vignette elicited a score suggesting an increase in dose. Clinic visits provide opportunities to observe and ensure that patients are provided comprehensive individualized treatment, and perhaps clinic visits in a clinical trial have less influence on short-term lapse than clinic visits have on treatment response in an office-based physician MOUD practice. That clinic visit was ranked last among the four treatment variables examined in our analysis of clinical trial participants suggests that the other components of individualized treatment in office-based treatment may have greater effectiveness than currently considered.

We acknowledge there may be differences in patient characteristics and treatment protocols between the clinical trials and the recent office-based practice survey [41]. We also acknowledge that our modeling of multiple treatment factors may not yet reflect the complexity of MOUD in terms of the specifications of modeling relations between treatment variables or between treatment variables and patient covariates, including co-occurring disorders. The results from our analysis of treatment factors suggest that, in addition to personalized buprenorphine dosing, increasing therapeutic engagement may result in reduced lapse and relapse due to more opportunities to evaluate patients, adapt MOUD, and encourage retention. Increases in therapeutic engagement may be possible for some waivered physicians if practice-related challenges are addressed [9,43].

### 4.4. Research Implications

Each of the treatment factor findings suggests future research hypotheses to be explored in this or similar datasets of individual patient data from buprenorphine MOUD, for example, the distribution of time-weighted dose supports the range of doses recommended by clinical experts, guidelines and the FDA (a maximum daily dose of 24 mg buprenorphine) [21,44]. The protective effects of dose and adaptive dose support suggestions that doses greater than those necessary to prevent opioid withdrawal symptoms are needed for maintenance treatment [18]. Additional analyses of dose, craving and withdrawal symptoms, lapse and relapse, are needed to evaluate whether there are additional benefits to increasing buprenorphine daily dose. The identification of more subtle relations between dose, lapse, relapse, duration of and retention in treatment will be assisted by the availability of future CTN trial data [45]. The moderation of treatment variable effects by analyses of addiction severity [46], specific comorbidities [39,40] or demographic and clinical characteristics [10] may help address the complexities of buprenorphine MOUD.

The identification of factors that influence non-pharmacologic treatment effects is another research priority, that is, the three treatment factors other than buprenorphine dose include therapeutic interactions. Each interaction could be further examined using physician or clinical staff characteristics, engagement metrics, and patient characteristics to improve understanding of how these influence treatment outcomes. Beyond additional analyses of individual treatment variables and patient covariates, lies the more complex effort of understanding which combination of treatment and patient factors results in long-term functional restoration. An analysis challenge will be to locate datasets of buprenorphine MOUD with such detail.

## 5. Conclusions

Our analysis of publicly available data from 3022 individuals randomized or enrolled into buprenorphine maintenance treatment for DSM-IV opioid dependence identifies, ranks and interprets significant effects of four novel time-dependent treatment predictors: dose, adaptive dose, time-in-trial and clinic visit. Treatment variable modeling resulted in findings that align with clinical MOUD practice (dose maintenance, increasing therapeutic engagement) and that suggest exploration of the relative effectiveness of these two critical aspects of MOUD. Research implications include modeling medication dose, treatment engagement and patient covariate effects using approaches that analyze these factors in their complex relations with patient outcomes.

## Figures and Tables

**Table 1:** Treatment and Outcome Variables in N = 3022 Participants.

Variable	Input	Coding	Mean	Median	Range
Time Weighted Dose (mg)	Dose (mg)	Equation (1)	11.9	12	0–32
Time Weighted Adaptive Dose	Fixed or per clinician	Equation (1)	0.76	1.00	0–1
Time Weighted Clinic Visit	At home or in clinic	Equation (1)	0.34	0.03	0–1
Time-in-Trial (days)	Day of clinic visit	Assigned	112.7	87	1–527
Short-term Lapse (Outcome) *	Urinalysis or self-report	Positive = 1	0.41	0	0–1

* Positive urinalysis or self-report, without reference to prior negative or positive tests or self-reports.

**Table 2:** Standardized Time Dependent Treatment Variable Effects on Short-term Lapse.

Variable	Effect *	SE **	95% CI	*p*-Value
Intercept	−0.056	0.146	(−0.341, 0.230)	0.702
Time Weighted Dose	−0.463	0.033	(−0.525, −0.400)	<0.001
Time Weighted Adaptive Dose	−0.354	0.039	(−0.430, −0.279)	<0.001
Time Weighted Clinic Visit	−0.209	0.035	(−0.278, −0.140)	<0.001
Time-in-Trial	−0.283	0.032	(−0.346, −0.221)	<0.001

* The estimated regression parameters obtained from fitting the general linear mixed model standard errors. ** Standard errors for the regression parameter estimates.

## Data Availability

Trial data are available from NIDA at datashare.nida.nih.gov, accessed on 5 September 2017.

## Supplementary Materials

The following supporting information can be downloaded at: https://www.mdpi.com/article/10.3390/ijerph19074106/s1, Table S1: Clinical Trial Study Designs; Table S2: Study Inclusion Criteria; Table S3: Study Exclusion Criteria I; Table S4: Study Exclusion Criteria II; Table S5: Demographics of Screened Individuals; Table S6: Sociodemographics of the Analysis Sample; Table S7: Drug Use History of the Analysis Sample; Table S8: Daily Dose, Urinalysis Days, and Time-Weighted Dose of a Single Participant.
