# Supplementary material for: Effects of Buprenorphine Dose and Therapeutic Engagement on Illicit Opiate Use in Opioid Use Disorder Treatment Trials
Source: Int J Environ Res Public Health. 2022 Mar 30;19(7):4106. doi: 10.3390/ijerph19074106 (PMC8998781; doi:10.3390/ijerph19074106)
Supplement: Supplementary file 1 [file ijerph-19-04106-s001.zip › ijerph-1641746-supplementary.pdf]

Supplementary Tables for "Effects of Buprenorphine Dose and Therapeutic Engagement on Illicit Opiate Use in Opioid Use Disorder Treatment Trials"

Andrew W. Bergen, James W. Baurley, Carolyn M. Ervin, Christopher S. McMahan, Joe Bible, Randall S. Stafford, Sesh C. Mudumbai, Andrew J. Saxon

---

List of Tables

1 Clinical Trial Study Designs . . . 3
2 Study Inclusion Criteria . . . 4
3 Study Exclusion Criteria I . . . 5
4 Study Exclusion Criteria II . . . 6
5 Demographics of Screened Individuals . . . 7
6 Sociodemographics of the Analysis Sample . . . 8
7 Drug Use History of the Analysis Sample . . . 9
8 Daily Dose, Urinalysis Days, and Time-Weighted Dose of a Single Participant 10

Table S1: Clinical Trial Study Designs

**Trial**	**CSP-999**	**CSP-1008A**	**CSP-1008B**	**CSP-1018**	**CTN-0027**	**CTN-0030**
Years, Start-End	'92-'97	'96-'97	'96-'97	'99-'02	'06-'10	'06-'09
Allocation	Randomized	Randomized	Non-randomized	Non-Randomized	Randomized	Randomized
Intervention	Parallel	Mixed	Single group	Single group	Parallel	Parallel
Masking	Double blind	Mixed	Open label	Open label	Open label	Open label
Phases	Two	Two	One	One	One	Two
Purpose	Safety/Efficacy	Efficacy/Safety$^{\alpha}$	Safety	Efficacy$^{\beta}$	Safety	Efficacy
Tx length max (mo)	12	12	12	12	8	8
Tx Phase One (wk)	40	4	52	52	24-32	4 Rx, 8 Counseling
Rx$^{\gamma}$ Phase One	B	B:B/N:P	B/N	B/N	B/N, M	B/N
Rx Dose (g/d) Phase One	1:4:8:16	16:16/4,P	Per physician	Per physician	Per physician	Per physician
Psychosocial Phase One	1-4 hr/mo	1 hr/wk	$\geq$ 1hr/wk	0.5 hr/wk	Per clinic	0.4:1.3 hr/wk$^{\delta}$
Administered Dose?	Observed	Mixed$^{\epsilon}$	Per physician	Not observed	Mixed$^{\zeta}$	Not observed
Tx Phase Two (wk)	12	48	-	-	-	16
Rx Phase Two	B	B/N	-	-	-	B/N
Rx Dose Phase Two	Per physician	Per physician	-	-	-	Per physician
Psychosocial Phase Two	1 hr/wk	$\geq$ 1hr/wk	-	-	-	0.3:1.2 hr/wk
Administered Dose?	Observed	Per physician	-	-	-	Not observed
Urinalysis as Outcome	Primary$^{\eta}$	Primary	Secondary	Primary	Secondary	Primary

From `clinicaltrials.gov` and `datashare.nida.nih.gov`. $^{\alpha}$For Phase One, and Safety for Phase Two. $^{\beta}$For adult participants, and safety, efficacy, and feasibility of detoxification, then maintenance per clinical judgement in participants 15 to 20 years of age. $^{\gamma}$Rx: B=buprenorphine, B/N=buprenorphine/naloxone, P=placebo, M=methadone. $^{\delta}$Standard Medical Management *vs* Enhanced Medical Management, aka Opioid Drug Counseling). $^{\epsilon}$Doses are observed weekdays, take-home doses weekends. $^{\zeta}$For B/N, first three days observed, remaining doses per physician; for M, observed. $^{\eta}$Self-reported lapse.

Table S2: Study Inclusion Criteria

Study	Inclusion Criteria*
CSP-999	Males and females (non-pregnant, non-nursing), ages 21-50. Current opiate dependence according to DSM-III-R/DSM-IV criteria with daily use for past month. Agreeable to conditions of the study and signs informed consent. Resident within commuting distance of clinic. Urine negative for methadone.
CSP-1008A	Males and females (non-pregnant, non-nursing), ages 18-59 inclusive. Current opiate dependence according to DSM-IV criteria. Treatment seeking. Agreeable to conditions of the study and able to give informed consent.
CSP-1008B	Males and females (non-pregnant, non-nursing), ages 18-59 inclusive. Current opiate dependence according to DSM-IV criteria. Treatment seeking. Agreeable to conditions of the study and able to give informed consent.
CSP-1018	Males and females (non-pregnant, non-nursing), at least 15 years of age. Current opiate dependence according to DSM-IV criteria. Treatment seeking with the desire to discontinue opiate use as an initial goal but willing to consider and accept longer treatment if necessary. Be in good physical health, or in the care of a physician for medical or psychiatric treatment. Agreeable to conditions of the study and able to give informed consent.
CTN-0027	Males and females (non-pregnant, non-nursing), at least 18 years of age. Current opiate dependence according to DSM-IV-TR criteria. Treatment seeking. Agreeable to conditions of the study and able to give informed consent.
CTN-0030	Males and females at least 18 years of age, treatment seeking (detoxification), and meet DSM-IV criteria for current prescription opioid dependence ($\geq$ 20 days/month). Without chronic pain severe enough to require ongoing opioid therapy or an acute pain event within the past six months. Medically stable and psychiatrically stable in the opinion of the study investigator. Good general health, or, for patients receiving ongoing medical or psychiatric treatment, participant's physician must be willing to continue management and cooperate with study physicians. Participants receiving opioids for pain must have clearance from their prescribing physician to enter the trial; the study physician will consult to ensure that the participant is medically stable enough to enter the trial. Participants who use prescription opioids by injection may be included as long as they have never injected heroin. Agreeable to conditions of the study and able to give informed consent.

*From `clinicaltrials.gov` and `datashare.nida.nih.gov` protocols. Some rewording to enhance comparability.

Table S3: Study Exclusion Criteria I

Study	Exclusion Criteria*
CSP-999	Any acute medical condition that would make participation in the study medically hazardous for the patient, with extra monitoring and hepatologist consultation where liver enzymes are > 5 and > 8 times upper limit of normal, respectively. DSM-III-R diagnosis of current alcohol dependence or sedative-hypnotic dependence. Current daily use of anticonvulsants, Antabuse, or neuroleptics. Expected inability to complete study. Enrollment in a methadone maintenance program within past 30 days. Participation in another research project. Female of childbearing potential who refuses birth control.
CSP-1008A	Any acute medical condition that would make participation in the study medically hazardous for the patient. Liver enzyme levels > 3 times upper limit of normal. Current use of systemic anti-retroviral or anti-fungal therapy. DSM-IV diagnosis of any psychoactive substance dependence other than opiate, caffeine, or nicotine dependence. Licit or illicit use of methadone, LAAM or naltrexone within the last days. Use of buprenorphine, other than as an analgesic, within the last 365 days. Expected inability to complete study. Enrollment in a methadone or LAAM maintenance program within past 45 days. Female of childbearing potential who refuses birth control.
CSP-1008B	Any acute medical condition that would make participation in the study medically hazardous for the patient. Liver enzyme levels > 3 times upper limit of normal. Current use of systemic anti-retroviral or anti-fungal therapy. DSM-IV diagnosis of any psychoactive substance dependence other than opiate, caffeine, or nicotine dependence. Licit or illicit use of methadone, LAAM or naltrexone within the last days. Use of buprenorphine, other than as an analgesic, within the last 365 days. Expected inability to complete study. Enrollment in a methadone or LAAM maintenance program within past 45 days. Female of childbearing potential who refuses birth control.
CSP-1018	Any acute medical condition that would make participation in the study medically hazardous for the patient, with hepatologist consultation where liver enzymes are > 8 times upper limit of normal. Known sensitivity to buprenorphine or naloxone. Acutely psychotic, severely depressed or an immediate suicide risk. Dependence upon alcohol, benzodiazepines or other drugs of abuse (excluding tobacco) requiring immediate medical attention. Participation in an investigational drug study within the past 30 days. Discontinued participation in a methadone or LAAM treatment program within the past 30 days. Licit or illicit use of methadone, LAAM or naltrexone for more than 30 days before enrolling in this study. Female of childbearing potential who refuses birth control. Expected inability to complete study.

*From `clinicaltrials.gov` and `datashare.nida.nih.gov` protocols. Some rewording to enhance comparability.

Table S4: Study Exclusion Criteria II

Study	Exclusion Criteria*
CTN-0027	Any acute medical condition that would make participation in the study medically hazardous for the patient. Liver enzymes (either) $>$ 5 times upper limit of normal. Alkaline phosphatase $>$ 3 times upper limit of normal. Model for Endstage Liver Disease score $\geq$ 11. Total bilirubin $\geq$ 2mg/dl, albumin $<$ 2.5 g/dl or prothrombin time $>$ 3 seconds. Participants with cardiac risk factors as confirmed by abnormal ECGs. Known sensitivity to buprenorphine, methadone, naloxone or any study medication ingredient. Diagnosis of acute psychosis, severe depression or an immediate suicide risk. DSM-IV dependence upon alcohol, benzodiazepines, other depressants or stimulants, requiring medical immediate attention or likelihood of intravenous misuse by past history of intravenous use. Participation in an investigational drug study within the past 30 days. Treatment with methadone, buprenorphine, buprenorphinie/naloxone, for $\geq$ 15 of the past 30 days (illicit use is not an exclusion). Female of childbearing potential who refuses birth control. Expected inability to complete study. Poor venous access.
CTN-0030	Any medical condition that would make participation in the study medically hazardous for the patient, in the opinion of the study investigator/physician, based on a review of medical records and baseline evaluations. Known sensitivity to buprenorphine, or naloxone. Diagnosis of acute psychosis, severe depression, or a suicide risk within the past 30 days. DSM-IV dependence upon alcohol, sedative-hypnotics or stimulants, and requiring medical immediate attention. Currently receiving formal substance abuse treatment. Have used heroin $\geq$ 4 days in the past 30 days. Lifetime opioid dependence accounted for by heroin use only. Have ever used heroin by injection. Have experienced a traumatic or major pain event within the past six months. Participation in another investigational drug study within the past 30 days. Participated in methadone or buprenorphine maintenance treatment for opioid dependence within 30 days of study enrollment. Female of childbearing potential who refuses birth control. Expected inability to complete study due to residence change or scheduled surgery. Pain of sufficient severity as to require ongoing pain management with opioids.

*From `clinicaltrials.gov` and `datashare.nida.nih.gov` protocols. Some rewording to enhance comparability.

Table S5: Demographics of Screened Individuals

Trial	CSP-999	CSP-1008A	CSP-1008B	CTN-1018	CTN-0027	CTN-0030	Overall
Totals	(N = 736)	(N = 449)	(N = 282)	(N = 596)	(N = 1920)	(N = 870)	(N = 4853)
Age (Years)							
Mean (SD)	36.0 (7.8)	37.6 (8.9)	40.0 (7.7)	35.8 (10.5)	37.4 (11.1)	33.2 (10.2)	36.33 (10.6)
Min, Max	(18, 67)	(19, 60)	(19, 59)	(15, 66)	(18, 68)	(18, 77)	(18, 77)
Missing (n, %)	2 (0.3)	123 (27.4)	77 (27.3)	29 (4.9)	651 (33.1)	216 (24.8)	1098 (22.6)
Gender, n (%)							
Male	497 (67.5)	290 (64.7)	202 (71.6)	394 (66.1)	1285 (67.0)	524 (60.3)	3192 (65.8)
Female	239 (32.5)	158 (35.3)	80 (28.4)	202 (33.9)	632 (33.0)	345 (39.7)	1656 (34.2)
Missing	0 (0.0)	1 (0.0)	0 (0.0)	0 (0.0)	3 (0.1)	1 (0.0)	5 (0.1)
R/Eth, n (%)							
W, Non-His	359 (48.8)	257 (57.4)	115 (40.8)	437 (73.4)	1243 (64.7)	751 (86.3)	3162 (65.2)
B, Non-His	161 (21.9)	136 (30.4)	94 (33.3)	46 (7.7)	212 (11.0)	30 (3.4)	679 (14.0)
Nat Amer	5 (0.7)	4 (0.9)	0 (0.0)	7 (1.2)	65 (3.4)	16 (1.8)	97 (2.0)
As/Pac Is	4 (0.5)	8 (1.8)	2 (0.7)	11 (1.8)	36 (1.9)	4 (0.5)	65 (1.3)
Hispanic	207 (28.1)	43 (9.6)	71 (25.2)	94 (15.8)	329 (17.1)	42 (4.8)	786 (16.2)
Other	0 (0.0)	0 (0.0)	0 (0.0)	0 (0.0)	35 (1.8)	27 (3.1)	62 (1.3)
Missing	0 (0.0)	1 (0.2)	0 (0.0)	1 (0.2)	0 (0.0)	0 (0.0)	2 (0.004)

Table S6: Sociodemographics of the Analysis Sample

Characteristic	N or Mean	% or SD
**Age**	36.1	9.8
**Gender**		
Men	2017	67
Women	1005	33
**OMB Race/Ethnicity**		
Other	6	<1
Asian	48	2
American Indian	50	2
Black	422	14
Hispanic	495	16
White	2001	66
**Education**		
Less than 7th grade	15	<1
Complete Graduate School	89	3
Junior High School	116	4
Standard College	213	7
Partial High School	304	10
Partial College	829	27
High School	1456	48
**Employment Type**		
Military	1	<1
Controlled	17	1
Student	64	2
Retired	84	3
Regular PT	232	8
Irregular PT	284	9
Unemployed	582	19
Fulltime	1758	58
**Employment History**		
Executive	53	2
Business Manager	101	3
Unskilled	235	8
Adminstrative	239	8
Clerical/Sales	407	13
Machine Operator	445	15
Never Gainfully	653	22
Skilled Manual	889	29
**Marital Status**		
Remarried	20	<1
Widowed	39	1
Seperated	261	9
Divorced	602	20
Never Married	1014	34
Married	1038	34
**Living Arrangement**		
No Stable	24	1
Controlled	25	1
Child Only	183	6
Alone	190	6
Friends	255	8
Family	263	9
Parents	294	10
Partner Only	537	18
Partner and Child	1251	41
**Annual Income**	$20,834	$30,024

Table S7: Drug Use History of the Analysis Sample

Variable	N or Mean	% or SD
**Heroin and opioid use**		
Heroin use	2354	78
No heroin use	668	22
**Years of opiate/opioid use**	8.23	8.41
**Primary mode of opiate/opioid use**		
Oral	122	4
IV	1710	57
Snort	1089	36
Smoking	74	2
Sublingual	5	$< .01$
Other	22	1
**Cocaine use**		
YES	1837	61
NO	1185	39
**Methamphetamine use**		
YES	718	24
NO	2304	76
**Alcohol use**		
YES	1891	63
NO	1131	37
**Tranquilizer use**		
YES	1025	34
NO	1997	66
**Marijuana use**		
YES	1953	65
NO	1069	35
**Phencyclidine use**		
YES	477	16
NO	2545	84

Table S8: Daily Dose, Urinalysis Days, and Time-Weighted Dose of a Single Participant

Variable	Data
Daily Dose	8 16 24 24 32 32 32 32 32 32 32 32 32 32 32 32 32 32 32 32 32 32 32 32 32 32 32 32 32 32 32 32 32 32 32 32 32 32 32 0 0 32 32 32 32 32 32 32 32 32 32 32 32 32 32 32 32 32 32 32 32 32 32 32 32 32 0 32 32 32 32 0 32 32 32 32 32 32 32 32 32 32 32 0 32 32 32 32 32 32 0 32 32 0 32 32 32 32 32 32 32 32 32 32 32 32 32 32 30 30 30 30 30 30 30 30 32 30 30 30 30 0 32 30 30 30 30 30 30 30 30 30 30 30 30 30 30 30 30 30 30 30 30 30 30 30 30 30 30 26 26 26 28 28 28 28 28 28 24 26 26 26 26 22 0 24 24 24 24 24 24
Urinalysis Days	7 17 23 35 41 52 56 64 72 79 93 101 108 122 128 136 143 149 158 163 171
Time Weighted Dose	29.58730 31.99768 31.99996 32.00000 16.00000 31.97656 31.99854 31.99999 31.00000 31.74219 23.93748 31.71851 31.99780 30.06274 29.59473 29.99842 29.99999 30.00000 27.95312 25.99854 23.62499
